# Physiological glomerular filtration rate and responses to Zeitgeber time and cold stress in C57BL/6 mice

**DOI:** 10.1080/0886022X.2026.2648347

**Published:** 2026-04-06

**Authors:** Xinran Wang, Yanqi Liu, Yu Xin, Hongxu Li, Lifeng Shen, Yu Xiao, Yuchen Song, Linqiong Liu, Kaijiang Yu, Changsong Wang

**Affiliations:** ^a^ Department of Critical Care Medicine, The First Affiliated Hospital of Harbin Medical University, Harbin, China; ^b^ Department of Critical Care Medicine, The Fourth Affiliated Hospital of Harbin Medical University, Harbin, China; ^c^ Department of Critical Care Medicine, Hainan Hospital of Chinese People’s Liberation Army General Hospital, Sanya, China

**Keywords:** Glomerular filtration rate, transcutaneous GFR measurement, C57BL/6 mice, zeitgeber time, cold stress

## Abstract

Transcutaneous FITC-sinistrin clearance provides a minimally invasive method to measure real-time glomerular filtration rate (GFR) in small animals. However, large-scale physiological reference data and analyses of time-of-day and environmental temperature influences remain limited. We aimed to establish reference values for GFR in healthy C57BL/6 mice and to assess variations related to Zeitgeber timing and cold stress. A total of 200 male mice were studied using a transcutaneous GFR monitoring system under standard conditions, at four Zeitgeber points (ZT2, ZT8, ZT14, ZT18), and following exposure to 4 °C for 2 or 4 h. Baseline GFR was 1.215 ± 0.201 mL/min/100 g body weight. Four-hour cold exposure increased GFR (1.129 ± 0.207 to 1.541 ± 0.333, *p* = 0.0004), while 2-hour exposure showed a non-significant trend (*p* = 0.0795). No significant differences were observed among Zeitgeber timepoints (*p* = 0.5317), though slightly elevated levels were noted during the active (dark) phase. This study provides baseline GFR reference values for healthy C57BL/6 mice and indicates that GFR is responsive to cold stress but remains relatively stable across the light–dark cycle, offering essential data for future renal physiology and experimental nephrology research.

## 1. Introduction

Glomerular filtration rate (GFR) serves as a pivotal measure of renal function and is extensively utilized in the diagnosis, classification, and progression of kidney diseases. In animal models of renal injury, real-time GFR monitoring is a critical component. Inulin, once administered to the body, is completely filtered by the glomerulus without secretion or reabsorption by the renal tubules, making it an ideal marker for measuring GFR. However, the measurement process is complex and invasive, as it requires repeated blood and urine collection from experimental animals in a short time frame [1]. The frequent invasive procedures required by traditional methods for measuring GFR necessitate deep anesthesia in small laboratory animals. Moreover, deep anesthesia can affect both hemodynamics and GFR [2]. To reduce the invasiveness of procedures and simplify GFR measurement, a minimally invasive technique using a micro-optical device and the exogenous renal marker fluorescein isothiocyanate-sinistrin (FITC-sinistrin) has been developed for real-time transcutaneous GFR monitoring [1,3,4]. FITC-sinistrin is freely filtered by the glomerulus, and its renal tubular reabsorption and secretion are negligible. After intravenous injection into mice, an optical device placed on the mouse’s skin excites the fluorescence of the tracer, which is emitted through the skin and collected to generate a FITC-sinistrin elimination curve. This method allows for real-time reflection of GFR in mice [1,5].

Numerous factors influence GFR, including light-dark cycles and environmental temperature variations [6–8]. In rodents, renal function varies across the 24-h circadian cycle, a pattern often described in terms of Zeitgeber time (ZT), which refers to time points relative to lights-on (ZT0) and lights-off (ZT12). Previous studies suggest that GFR peaks during the active phase, especially near ZT16, and is modulated by the intrinsic molecular clock of podocytes [9,10]. However, transcutaneous GFR data under differing Zeitgeber phases remain scarce.

Cold exposure is another environmental factor that can influence GFR. Acute cold stress may alter renal hemodynamics and tubular function, possibly *via* reduced metabolic demands or neuroendocrine responses [8]. Yet, how cold stress modifies transcutaneously measured GFR remains insufficiently explored.

Our research team focuses on acute kidney injury (AKI) and has developed multiple murine models. We have accumulated a large dataset of transcutaneously measured GFR values. Given the limited availability of transcutaneous GFR data across different Zeitgeber phases and under cold stress conditions, we hypothesized that baseline GFR in healthy C57BL/6 mice may exhibit Zeitgeber time–dependent variation under physiological, conscious conditions, and that acute cold exposure would induce a transient but measurable alteration in GFR dynamics.

Accordingly, the aim of this study was to systematically characterize physiological GFR values and to evaluate their responses to selected Zeitgeber time points (ZT2, ZT8, ZT14, and ZT18) and acute cold stress (4 °C for 2 or 4 h) using a noninvasive transcutaneous FITC-sinistrin approach, thereby providing benchmark reference data for future experimental renal research.

## 2. Methods

### 2.1. Animals

A total of 200 healthy male C57BL/6 mice (8–12 weeks old; Charles River Laboratories, Beijing) were used in this study. GFR measurements were obtained only from baseline data of animals without any signs of illness, prior interventions, or environmental stress exposure. The mice were housed under SPF conditions in a controlled environment with a 12-h light/dark cycle (lights on 07:00 [ZT0], off 19:00 [ZT12]), maintained at a temperature of 25 ± 1 °C and humidity of 55 ± 2%, with *ad libitum* access to food and water. All procedures involving animal anesthesia and euthanasia adhered to institutional and international guidelines. Mice were anesthetized with avertin prior to terminal procedures, and euthanasia was performed by cervical dislocation in accordance with the Animal Euthanasia Guidelines (2020). All animal procedures were approved by the Institutional Animal Care and Use Committee of the First Affiliated Hospital of Harbin Medical University (Ethical Review of the Use and Welfare of Laboratory Animals, Approval No. IACUC: 2023022).

### 2.2. GFR measurement method

**Figure 1.**
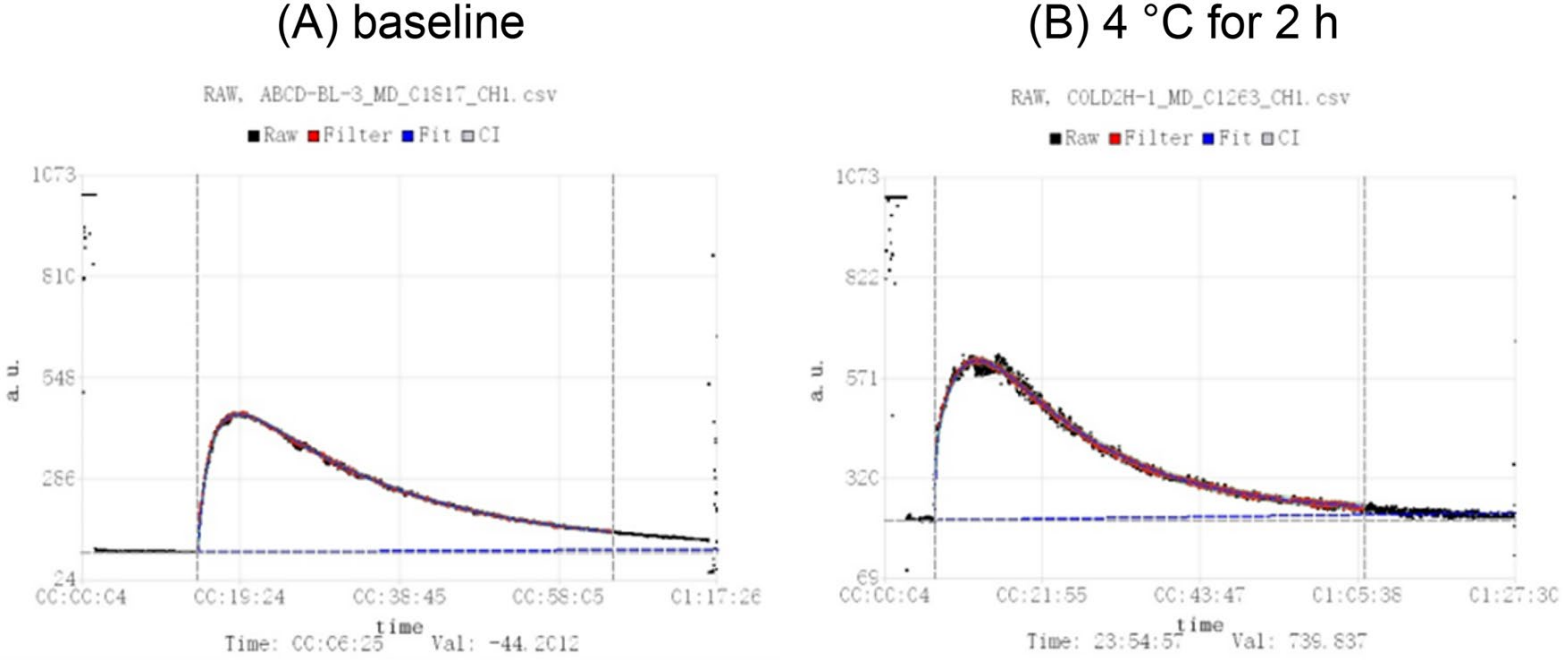
FITC-sinistrin clearance curves under baseline and cold exposure conditions. (A) Baseline; (B) Cold exposure (4 °C, 2 h).

GFR was quantified using a previously validated transcutaneous real-time measurement method [3,11] with a transcutaneous monitoring system (MediBeacon GmbH, Mannheim, Germany). FITC-sinistrin (MediBeacon GmbH, Mannheim, Germany) measurement is based on its property of being freely filtered by the glomeruli without tubular reabsorption or secretion. After intravenous injection of FITC-sinistrin *via* the tail vein, the tracer gradually enters the circulation. An optical probe, fixed to the shaved right dorsal skin, emits excitation light at a specific wavelength and continuously detects the emitted fluorescence signal. In accordance with the MediBeacon system, the algorithm automatically excludes the initial distribution phase after injection, and only the elimination phase is used for curve fitting and GFR calculation. The raw fluorescence intensity–time data (black) are filtered to reduce noise (red), and the elimination phase is fitted with a decay model (blue), with the shaded area representing the 95% confidence interval. The elimination half-life (t_1_/_2_) was obtained from the fitted clearance curve and converted to GFR using the system’s standard calculation algorithm. Figure 1A shows a representative clearance curve under baseline conditions, and Figure 1B depicts a curve obtained after 4 °C cold exposure for 2 h.

To assess diurnal variation in renal function, GFR measurements were obtained at four Zeitgeber timepoints (ZT2, ZT8, ZT14, and ZT18).Previous studies have demonstrated time-dependent fluctuations in renal function across different Zeitgeber phases [10,12]. Measurements were conducted over a 6-month period across multiple batches, using seven transcutaneous monitoring devices in parallel. All devices were regularly calibrated, and quality control was performed throughout the study. No significant inter-device heterogeneity was detected, ensuring comparability and consistency of GFR results across the entire cohort. Measurements with unsuccessful FITC-sinistrin tail vein injections (e.g., extravasation, failed venous access) or with motion artifacts leading to signal loss were excluded and repeated on the following day.

### 2.3. Cold stress exposure

Two groups of mice (*n* = 15 each) were placed in a 4 °C chamber for either 2 or 4 h. GFR was measured at room temperature immediately before and after cold exposure. During cold exposure, mice had access to food and water and were housed in bedding-lined cages to prevent excessive hypothermia.

### 2.4. Zeitgeber time measurement

To evaluate GFR variation by time of day, measurements were performed at ZT2 (9:00 AM), ZT8 (3:00 PM), ZT14 (9:00 PM) and ZT18 (1:00 AM) in 18 mice. Measurements were separated by approximately 4–6 h to allow for full tracer clearance.

### 2.5. Statistical analysis

Data are presented as mean ± standard deviation (SD). One-way ANOVA was used to compare GFR across Zeitgeber timepoints. For cold stress experiments, paired t-tests were used to assess pre- and post-exposure differences. A *p* value < 0.05 was considered statistically significant. All analyses were conducted using GraphPad Prism v10.4 (GraphPad Software, San Diego, CA, USA).

## 3. Results

### 3.1. GFR in male C57BL/6 mice

**Figure 2.**
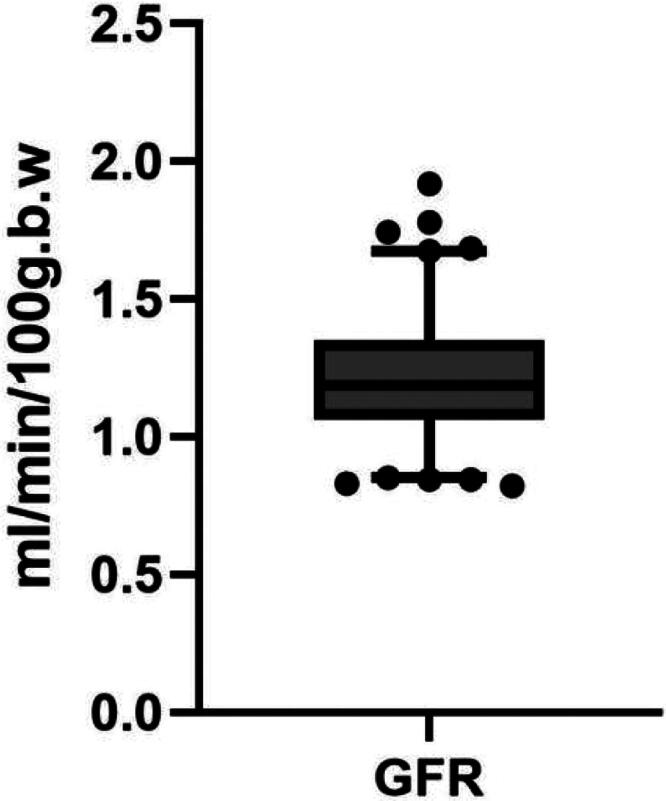
Distribution of physiological GFR in male C57BL/6 mice. Box-and-whisker plots summarize GFR values from 200 mice; superimposed dots represent a subset of individual measurements shown for visualization only.

GFR in male C57BL/6 mice (*n* = 200) was determined using transcutaneous real-time monitoring technology, yielding a value of 1.215 ± 0.201 mL/min/100 g body weight; the overall distribution of GFR values is summarized in Figure 2.

### 3.2. Cold stress elevates GFR

**Figure 3.**
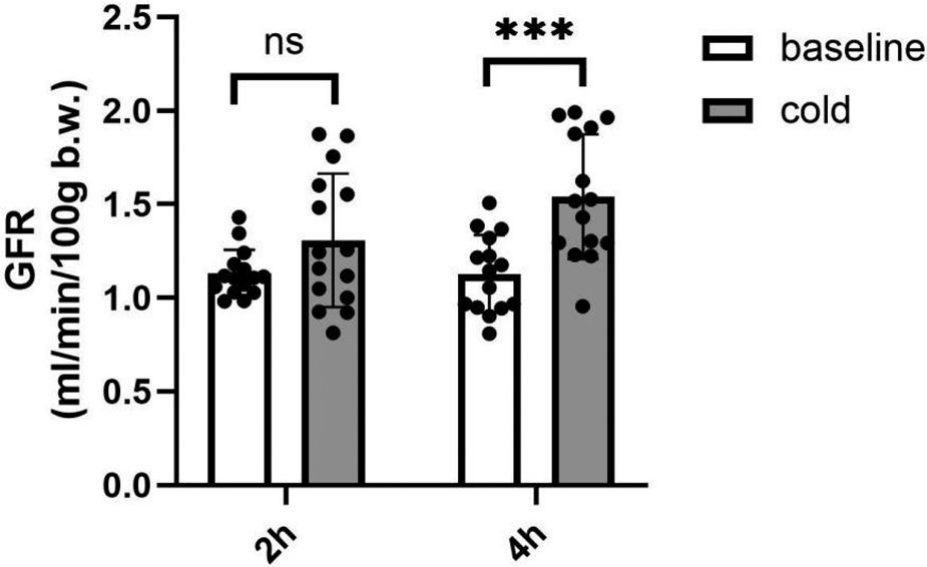
Cold stress-induced changes in GFR. **p < 0.01, ***p < 0.001

Cold exposure for 2 h led to an increase from 1.130 ± 0.127 to 1.308 ± 0.356 (*p* = 0.0795), while 4-h exposure raised GFR from 1.129 ± 0.207 to 1.541 ± 0.333 mL/min/100 g body weight (*p* = 0.0004). Results are shown in Table 1 and Figure 3.

**Table 1.** Cold stress-Induced Changes in GFR.

	GFR, mL/min/100 g body weight	*t*	*p*
***2 h***			
Baseline (*n* = 15)	1.130 ± 0.127	1.820	0.0795
4 °C 2 h (*n* = 15)	1.308 ± 0.356		
***4 h***			
Baseline (*n* = 15)	1.129 ± 0.207	4.066	< 0.01
4 °C 4 h (*n* = 15)	1.541 ± 0.333		

t, statistic from paired t-test; p, probability value.

Baseline (2 h) vs. Baseline (4 h): two-sample t-test, *p* = 0.0795.

### 3.3. Zeitgeber time does not significantly alter GFR

**Figure 4.**
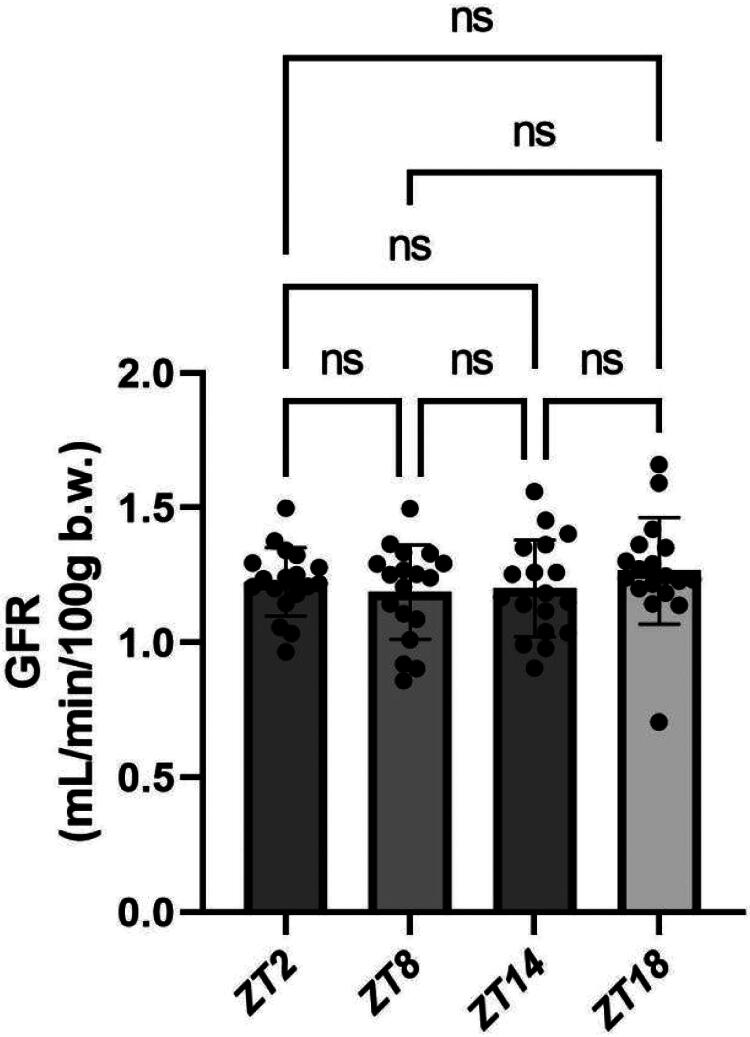
Zeitgeber-time–dependent variations in GFR.

GFR values at ZT2, ZT8, ZT14 and ZT18 were 1.226 ± 0.126, 1.187 ± 0.175, 1.202 ± 0.178 and 1.267 ± 0.198 mL/min/100 g body weight, respectively (*F* = 0.740, *p* = 0.5317).A modest upward trend was observed during the dark (active) phase (ZT14–ZT18), although it was not statistically significant (Figure 4 and Table 2).

**Table 2.** Zeitgeber-time–dependent variations in GFR.

Zeitgeber Time	GFR, mL/min/100 g body weight	*F*	*p*
ZT2 (*n* = 18)	1.226 ± 0.126	0.740	0.5317
ZT8 (*n* = 18)	1.187 ± 0.175		
ZT14 (*n* = 18)	1.202 ± 0.178		
ZT18 (*n* = 18)	1.267 ± 0.198		

F: statistic from one-way ANOVA; p: probability value.

## 4. Discussion

In this study, we applied a noninvasive transcutaneous FITC-sinistrin approach to assess physiological GFR in conscious C57BL/6 mice. The principal findings are that (i) using a large sample, we provide benchmark reference values and a well-characterized distribution range of physiological GFR, helping to address the limitation of prior reports based on very small sample sizes; (ii) acute cold exposure induces a transient increase in GFR; and (iii) across selected Zeitgeber timepoints, GFR shows only modest variation that does not reach statistical significance despite a trend toward higher values during the early dark phase.

Transcutaneous measurement of GFR using the fluorescent dye FITC-sinistrin has emerged as a highly effective method for assessing renal function in conscious animals [13]. This minimally invasive, rapid, and convenient technique provides real-time data on kidney function. Several previous studies have employed this approach in small cohorts of mice, typically with sample sizes ranging from 6 to 10 animals [1,5,11,13]. In contrast, our study is the first large-scale report analyzing GFR data from 200 healthy male C57BL/6 mice using FITC-sinistrin transcutaneous real-time measurements.

Our results showed that the physiological GFR in C57BL/6 mice was 1.215 ± 0.201 mL/min/100 g body weight. This value is consistent with those reported in previous studies using the same technique in smaller cohorts, such as 1.2 ± 0.1 mL/min/100 g body weight (*n* = 7) [1]. Compared with these reports, our dataset, based on a substantially larger sample size, offers a more reliable and generalizable reference for physiological GFR. The observed range of baseline GFR values is consistent with the expected biological variability among healthy animals [1], which may result from minor inter-individual differences in body weight, metabolic rate, and renal physiology.

Our study also demonstrated a significant elevation in GFR following 4-h cold exposure. One plausible mechanism is cold-induced diuresis, in which vasoconstriction during cold stress enhances central blood volume and venous return, elevating central venous pressure and cardiac output [14]. This cascade may suppress antidiuretic hormone release *via* baroreceptor pathways, thereby reducing renal water reabsorption and promoting diuresis [15]. These hemodynamic changes may transiently increase glomerular filtration.

A subtle trend of higher GFR during the early dark phase (ZT14–ZT18) was noted compared with the light phase (ZT2–ZT8); however, the variation was small and did not reach statistical significance, consistent with the modest amplitude of physiological GFR oscillation reported in mice. In rodents, renal function varies across the 24-h circadian cycle, a pattern often described in terms of Zeitgeber time (ZT), which refers to time points relative to lights-on (ZT0) and lights-off (ZT12) [10]. Daily rhythmicity in renal function—driven by fluctuations in glomerular hemodynamics and tubular transport—and its regulation by circadian genes such as *Clock*, *Bmal1*, and *Per1* have garnered increasing attention [6,16]. Sympathetic nervous system activity and water–salt homeostasis also follow circadian patterns, and multiple rhythm-related genes are expressed in the kidney, potentially modulating GFR [17].

Differences in methodology across studies should be taken into account when interpreting diurnal variation in renal function. Earlier studies evaluating renal function or GFR-related parameters mainly employed inulin- or sinistrin-based plasma clearance methods, which differ from the noninvasive transcutaneous FITC-sinistrin approach used in the present study under conscious and physiological conditions. These methodological differences may partly account for the less pronounced time-of-day variation observed. Moreover, previous reports that described more marked diurnal differences did not normalize GFR to body weight, while our study expressed GFR as mL/min/100 g body weight to account for inter-animal variability; this normalization may smooth inter-timepoint variation and reduce the apparent amplitude of rhythmicity. Previous reports indicate that renal circadian rhythmicity in mice is relatively modest under physiological conditions, which may help explain the absence of statistically significant time-dependent differences in our findings.

This study has several limitations. First, although four Zeitgeber timepoints were included, more frequent sampling may be required to fully characterize the GFR rhythm across the 24-h cycle. Second, only male C57BL/6 mice were included. This design choice was made to minimize potential variability associated with the estrous cycle and to facilitate the establishment of stable physiological GFR reference values. Future studies incorporating female mice will be important to further explore potential sex-related differences in renal functional responses. Third, while potential mechanisms for cold-induced GFR changes were discussed, we did not directly measure relevant physiological or hormonal parameters, which limits mechanistic interpretation and should be addressed in future studies.

## Conclusion

The physiological GFR of male C57BL/6 mice, assessed *via* the transcutaneous FITC-sinistrin technique, was 1.215 ± 0.201 mL/min/100 g body weight. GFR increased following cold stress (4 °C for 4 h). These findings provide a robust physiological reference for future renal function studies in murine models.

## Data Availability

The authors confirm that the data supporting the findings of this study are available within the article and/or its supplementary materials.
